# Viral reactivations and co-infections in COVID-19 patients: a systematic review

**DOI:** 10.1186/s12879-023-08117-y

**Published:** 2023-04-26

**Authors:** Jenny Yeon Hee Kim, Martin Ragusa, Fernando Tortosa, Ana Torres, Lionel Gresh, Jairo Andres Méndez-Rico, Carlos Arturo Alvarez-Moreno, Thiago Costa Lisboa, Sandra Liliana Valderrama-Beltrán, Sylvain Aldighieri, Ludovic Reveiz

**Affiliations:** 1 Knowledge Translation Program, Evidence and Intelligence for Action in Health Department, Pan American Health Organization, Washington, DC USA; 2 Incident Management System for the COVID-19 Response, Pan American Health Organization, Washington, DC USA; 3 Department of Health Emergencies, Pan American Health Organization, Washington, DC USA; 4 Facultad de Medicina, Universidad Nacional de Colombia, Bogotá, Colombia; 5 Critical Care Department, Hospital de Clinicas de Porto Alegre, PPG Ciencias Pneumologicas, Universidade Federal do Rio Grande do Sul, UFRGS, Porto Alegre, Brazil; 6 Ph.D. Program in Clinical Epidemiology, Department of Clinical Epidemiology and Biostatistics, Faculty of Medicine, Pontificia Universidad Javeriana, Bogotá, Colombia; 7 Internal Medicine Department, Hospital Universitario San Ignacio, Pontificia Universidad Javeriana, Bogotá, Colombia

**Keywords:** Systematic review, Viral reactivation, Viral co-infection, COVID-19, Patient characteristics

## Abstract

**Background:**

Viral reactivations and co-infections have been reported among COVID-19 patients. However, studies on the clinical outcomes of different viral reactivations and co-infections are currently in limit. Thus, the primary purpose of this review is to perform an overarching investigation on the cases of latent virus reactivation and co-infection in COVID-19 patients to build collective evidence contributing to improving patient health. The aim of the study was to conduct a literature review to compare the patient characteristics and outcomes of reactivations and co-infections of different viruses.

**Methods:**

Our population of interest included confirmed COVID-19 patients who were diagnosed with a viral infection either concurrently or following their COVID-19 diagnosis. We extracted the relevant literature through a systematic search using the key terms in the online databases including the EMBASE, MEDLINE, Latin American Caribbean Health Sciences Literature (LILACS), from inception onwards up to June 2022. The authors independently extracted data from eligible studies and assessed the risk of bias using the Consensus-based Clinical Case Reporting (CARE) guidelines and the Newcastle–Ottawa Scale (NOS). Main patient characteristics, frequency of each manifestation, and diagnostic criteria used in studies were summarized in tables.

**Results:**

In total, 53 articles were included in this review. We identified 40 reactivation studies, 8 coinfection studies, and 5 studies where concomitant infection in COVID-19 patients was not distinguished as either reactivation or coinfection. Data were extracted for 12 viruses including IAV, IBV, EBV, CMV, VZV, HHV-1, HHV-2, HHV-6, HHV-7, HHV-8, HBV, and Parvovirus B19. EBV, HHV-1, and CMV were most frequently observed within the reactivation cohort, whereas IAV and EBV within the coinfection cohort. In both reactivation and coinfection groups, patients reported cardiovascular disease, diabetes, and immunosuppression as comorbidities, acute kidney injury as complication, and lymphopenia and elevated D-dimer and CRP levels from blood tests. Common pharmaceutical interventions in two groups included steroids and antivirals.

**Conclusion:**

Overall, these findings expand our knowledge on the characteristics of COVID-19 patients with viral reactivations and co-infections. Our experience with current review indicates a need for further investigations on virus reactivation and coinfection among COVID-19 patients.

**Supplementary Information:**

The online version contains supplementary material available at 10.1186/s12879-023-08117-y.

## Introduction

Viral reactivation is associated with different types of stimuli including physiologic and physical changes, and in particular, immunosuppression [1–3]. Viral reactivation consists of two cycles, including lytic and latent phases. Within lytic cycle, replication and expression of a viral genome and release of virions result in the lysis of host cells whereas during latency, some human viruses may remain dormant within host cells and establish persistent infection with limited or no production of viral particles [1–3]. The pathogens may switch between the latent and lytic cycles, and a process in which a latent virus enters the lytic stage is known as reactivation [1–3].

Patients with severe COVID-19 have been characterized by impaired immunity, hyperinflammation, lymphopenia, and cytokine storms [4]. Decline in a number of CD4+ and CD8+ T cells due to either direct attack from SARS-CoV-2 through the spike (S) protein binding with a receptor on T cell [4–7] or induced cellular apoptosis and the subsequent decline in the type I interferons, especially interferon-gamma (IFN-γ) [4, 8, 9], as well as the T cell exhaustion marked by the increased expression of programmed death 1 (PD-1) indicate the state of immunosuppression in COVID-19 patients [4, 10]. Moreover, elevated levels of IL-2 and TNF-α can mediate T cell apoptosis by promoting Fas signaling and exacerbate lymphopenia in COVID-19 patients [4, 11–13]. An impaired host immune system without a normal suppression of virus replication, consequently, may induce the reactivation of latent viruses in host, which could contribute to some of the neurologic, dermatologic, and hematologic manifestations among others in COVID-19 patients [14–24].

Adverse clinical outcomes from the interplays between SARS-CoV-2 and other respiratory and systemic viruses have also become evident from their synergistic impact on further increase of the inflammatory cells to the site of infection and elevation of proinflammatory cytokines [25–27]. For example, patients with SARS-CoV-2 and influenza virus coinfections were found to develop hyperinflammation, ARDS, myocarditis, acute kidney injury, and other disorders due to more frequent activation of the cytokine cascade by flu infection [28–32]. A retrospective study by Yue et al. revealed that compared to patients with COVID-19 alone, those coinfected with SARS-CoV-2 and Influenza B virus (IBV) were more likely to have poorer prognosis marked by the fatigue, abnormalities in chest computed tomography (CT), and decreased lymphocytes and eosinophils, however these findings were not consistent in patients with Influenza A Virus (IAV) [33]. Primary infection by a different virus, such as cytomegalovirus, followed by SARS-CoV-2 infection, may also predispose individuals to more severe COVID-19 by compromising immunity through disruption of T-cell differentiation and upregulation of interleukin-6 [28]. immunocompromised individuals could be more prone to viral coinfections as Lino et al. observed a higher prevalence of therapeutic immunosuppression status among HHV-6/SARS-CoV-2 coinfected patients compared to the SARS-CoV-2 patients only [34]. However, exact mechanisms by which secondary infections with viruses occur in COVID-19 patients are under investigation.

Studies have additionally reported the incidence of reactivated viruses in COVID-19 patients, mainly the *Herpesviridae* virus including herpes simplex type 1 and 2 (HSV-1 and HSV-2), varicella zoster virus (VZV), Epstein–Barr virus (EBV), cytomegalovirus (CMV), human herpes virus 6, 7 (HHV 6, 7) [14–24]. In rare cases, hepatitis B virus (HBV) reactivation in chronic patients have also been observed [35, 36]. le Bal’ch et al. reviewed the virology results of 38 COVID-19 patients and found the patients with either CMV or HSV reactivation required prolonged mechanical ventilation compared to patients with no reactivated *Herpesviridae* virus [15]. Similarly, Simonnet et al. performed systematic testing for EBV, CMV, HHV-6 DNAemia on critically ill COVID-19 patients and observed the virus reactivations in 85% of patients, among which the patients with EBV reactivation required longer hospital length-of-stay [17]. Skin and ocular manifestations, too, including lesions in multiple regions and herpetic keratitis with risk of blindness, have been observed in patients with *Herpesviridae* reactivations [18, 19]. Liu et al. reported HBV reactivation in three COVID-19 patients with a history of chronic HBV infection where an increase in HBV DNA upon admission was noted, with one patient developing cirrhosis [35].

Albeit the emerging evidence present a broad spectrum of medical complications among the patients, there is lack of knowledge on latent virus reactivation in COVID-19 cases. Published studies narrowly focus on specific virus species rather than conducting an overarching investigation on all relevant latent virus activation and coinfection in patients. To increase the effectiveness of complex patient care and prevent potential deterioration of patient condition from infections during COVID-19, it is critical to establish a solid understanding of the consequences of both virus reactivation and coinfections. Thus, through the systematic review, we attempted to investigate what were the reported clinical and demographic characteristics of patients undergoing either latent virus reactivation or coinfection, what diagnostic tools were used to detect the reactivation and coinfection, and whether any COVID-19 treatment was associated with an occurrence of either reactivation or coinfection, or both.

## Methods

This study was reported following the Preferred Reporting Items for Systematic Reviews and Meta-Analyses (PRISMA) 2020 statement (see Additional file 1 for the checklist) [37]. A predetermined study protocol of data sources, search strategies, inclusion criteria, and data extraction method was registered on the International Prospective Register of Systematic Reviews (PROSPERO, registration number: CRD42022340897) available at https://www.crd.york.ac.uk/prospero/display_record.php?ID=CRD42022340897.

### Population of interest

The primary population of interest consisted of all patients with confirmed COVID-19 diagnosis via reverse transcription polymerase chain reaction (RT-PCR), medical and admission history, or laboratory confirmed positivity if not specified otherwise, who were diagnosed with a viral infection either concurrently or following the COVID-19 diagnosis. There were no age restrictions.

### Data sources

We extracted the relevant literature through a systematic search using the key terms such as viruses, COVID-19, reactivation, latent infection, resurrection, coinfection in the online databases including the EMBASE, MEDLINE, Latin American Caribbean Health Sciences Literature (LILACS), from December 2019 up to June 2022. Manual search was also performed using the Google Scholar for identifying additional articles. Specific key terms for each database are listed in Appendix S1 in Additional file 2.

### Study selection

**Fig. 1:**
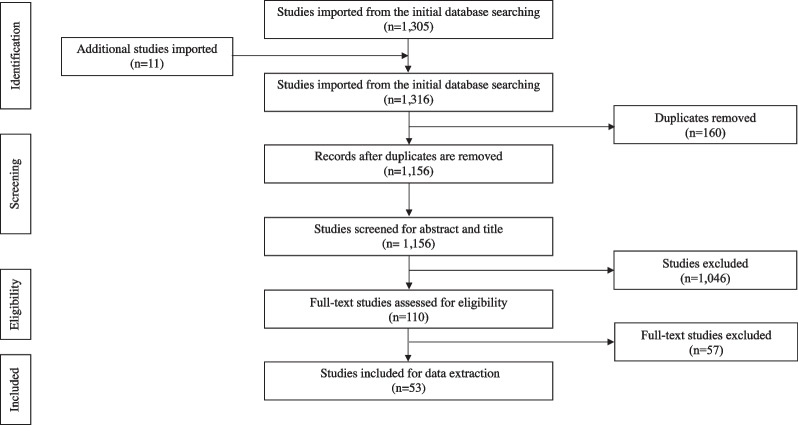
PRISMA flowchart

The review included cohort studies, cross-sectional studies, case reports, case series, preprints, and editorial letters with relevant cases described. Studies published in English, Portuguese, Spanish, French, and Korean were sought for. There were no restrictions on the country and age of the population. Studies targeting irrelevant population or animals, describing a case with suspected but no confirmed diagnosis without molecular testing, as well as the literature in the formats of commentary, survey, and recommendation were excluded from the review. Two investigators (YHK, LR) separately screened the titles and abstracts of articles and simultaneously resolved the conflicts (Fig. 1).

### Data extraction

We used MS Excel as a template for data extraction. Following information were extracted from each article: first author, publication year, study title, study site, study setting, study design, secondary infection type (reactivation, coinfection, not distinguished), total number of participants, total number of COVID-19 positive participants, total number of COVID-19 positive participants with secondary infection, age, sex/sex ratio, medical history and existing comorbidities, laboratory findings, clinical manifestations including symptoms and complications, secondary infection definition/criteria described in a study, diagnostic tools, treatments and procedures received.

### Risk of bias assessment

YHK, MT, FT, and MR independently assessed the risk of bias for cohort studies. YHK independently assessed the risk of bias for case reports. Meetings were arranged between authors to overlook and resolve any disagreements on the assessments. For the case reports and case series, the Consensus-based Clinical Case Reporting (CARE) guidelines will be used. For the cohort studies, the Newcastle–Ottawa Scale (NOS) will be used.

### Summarizing data

For cohort and cross-sectional studies that do not provide distinct clinical data by each virus, only the frequency of cases was imputed in tables. Laboratory findings summarized either in mean or median values were distinguished as categorical variables either as elevated or decreased levels using the external references [38–40] when the reference ranges were not provided. For each relevant laboratory variable, total reactivation cohort count was imputed. For case reports describing co-reactivations in a single patient, we added a count of 1 for variables for each virus type.

## Results

### Study selection

From the search, total 1316 records were extracted from searches. Upon removal of duplicates (n = 160), 1156 articles were eligible for title and abstract screening, among which 1046 were excluded due to irrelevant study settings, patient populations, and not having access to full texts. 110 records were eligible for the full text screening, among which 57 were excluded for insufficient data for the objectives of this review. Thus, 53 records reporting an infection secondary to COVID-19 diagnosis were included in this study (Fig. 1). All selected articles were published in either English or available with English-translated versions. No separate translation for other languages was required in data extraction.

### Study characteristics

Among 53 included studies, we found 22 case reports, 20 cohort studies, 7 case series, 2 cross-sectional studies, and 2 editorial letters in formats of 1 case report and 1 cohort study. Out of 45 studies based on inpatient settings, 24 studies reported the cases of infection in patients admitted to the Intensive Care Unit (ICU). There was 1 autopsy report, 1 Electronic Medical Record (EMR) based report, and 1 study with no specified study setting. The rest were based on ambulatory care settings (Table 1—located below the List of Abbreviations). Table 1 summarizes the characteristics of studies included in this review, including author and publication year, study site, study design and setting, total study population, age of study population expressed either in median or mean years, outcome of the study, COVID-19 diagnostic method, and diagnostic method for the outcome. Here, outcome refers to either viral co-infection or reactivation cases for which the authors provided the clinical outcomes of patients. Information on undetected viruses and patients with no additional information besides the infection status were excluded due to the nature of aims for this study.

**Table 1:** Study characteristics of selected records

Author (year)	Study site	Study design	Study setting	Total sample size	Age^a^	Outcome measure/Diagnosis of interest	COVID-19 Diagnostic method	Diagnostic method for outcome measure and relevant clinical outcomes^b^
Aldhaleei et al. (2020) [36]	United Arab Emirates	Case report	Inpatient (ICU)	1	36 years	COVID-19 induced HBV reactivation	RT-PCR	Blood tests (biochemistry, CBC, serology for HB core Ab, HBe Ag and Ab)
Aldehaim et al. (2022) [41]	Saudi Arabia	Case report	Inpatient (ICU)	1	47 years	CMV Pneumonitis in a COVID-19 patient with systemic lupus erythematosus and interstitial pneumonia	RT-PCR	Chest examination, CMV PCR, microscopy and staining, blood tests (CBC, biochemistry)
Ananthegowda et al. (2021) [42]	Qatar	Case report	Inpatient (ICU)	1	55 years	CMV meningoencephalitis in severe COVID-19 patients	RT-PCR	CMV CSF PCR, CSF analysis, Brain MRI, blood tests (CBC, biochemistry)
Carll et al. (2021) [43]	United states	Case report	Inpatient	1	Not reported	CMV hemorrhagic enterocolitis in severe COVID-19 patient	RT-PCR	Blood tests (biochemistry, CBC, CMV PCR), IHC
Das et al. (2022) [19]	India	Case report	Ambulatory care	1	73 years	Recurrent herpes simplex keratitis in a patient with a previous history of COVID-19 diagnosis	Tool not specified, based on the patient history	Ophthalmological exam, HSV-1 RT-PCR
Drago et al. (2021) [16]	Italy	Editorial letter (case report)	Ambulatory care	1	16 years	Pityriasis rosea (PR) diagnosis from the EBV, HHV-6, HHV-7 reactivations	RT-PCR	Blood tests (CBC, serology for all three viruses, including EBV IgM, EBV VCA, anti-VCA IgG, anti-early antigen IgG, anti EBV nuclear antigen IgG, HHV-6 and 7 IgM and IgG, RT-PCR for all three viruses, physical examination
Duong et al. (2021) [44]	Not reported	Case report	Inpatient	1	59 years	CMV induced acute hepatitis in COVID-19 patient	Tool not specified, based on medical records	CMV PCR, CBC
Ferreira et al. (2020) [45]	Brazil	Case report	Ambulatory care	1	39 years	VZV and COVID-19 co-infection	RT-PCR	Physical examination, brain MRI, blood tests (cytokine biochemistry, CBC, VZV IgM serology)
Figueredo et al. (2021) [46]	USA	Case report	Inpatient (ICU)	1	55 years	HBV reactivation in COVID-19 patient post tocilizumab therapy	Tool not specified, based on medical records	HBV PCR and serology for for HBV core Ab, HBe Ab, HBe Ag, HBV surface Ab, HBV surface Ag
Gardini et al. (2021) [47]	Italy	Case report	Inpatient (ICU)	1	61 years	Kaposi sarcoma in immunocompetent patient with SARS-CoV-2 infection	RT-PCR	Blood tests (CBC, biochemistry), neck ultrasound, CT scan, histologic examination, HHV-8 PCR
Ghobrial et al. (2021) [48]	USA	Case report	Inpatient (ICU)	1	72 years	CMV induced rectal ulcer in immunocompromised COVID-19 patient	Tool not specified, based on medical records	Biopsy and plasma CMV PCR
Gonzalez et al. (2021) [49]	Puerto Rico	Case report	Inpatient	1	32 years	HSV-2 induced ARN reactivation	RT-PCR	Ophthalmological exam, vitreous PCR for HSV-2, serology test for HSV-2 IgG
Librero et al. (2021) [50]	Spain	Case report	Inpatient	1	37 years	HBV reactivation in COVID-19 patient with a history of chronic HBV infection	Tool not specified, but indicated as laboratory confirmed	HBV PCR, blood test (biochemistry)
Khatib et al. (2021) [51]	Qatar	Case report	Inpatient	1	42 years	CMV colitis in SARS-CoV-2 patient on immunosuppressive treatment	RT-PCR	Blood test (cytokine, serology [not specified], biochemistry), CT scan, histologic examination
Magri et al. (2021) [52]	Italy	Case report	Inpatient	1	83 years	Kaposi Sarcoma in SARS-CoV-2 patient	NP swab, tool not specified	Physical examination, biopsy, histologic examination, serology test for HHV-8 antibodies (not specified)
Maillet et al. (2021) [53]	France	Case report	Inpatient (ICU)	1	75 years	CMV reactivation induced proctitis in an immunocompetent COVID-19 patient treated with immunomodulators	RT-PCR	Blood tests (biochemistry, CBC), CT scan, colonoscopy, stool culture, blood CMV PCR, rectal biopsy with immunohistochemistry staining
Mikami et al. (2021) [54]	France	Case report	Ambulatory care	1	28 years	CMV and parvovirus B19 infection in a SARS-CoV-2 IgG positive patient	RT-PCR, antibody test (tool not specified)	Physical examination, blood tests (serology for CMV-IgG, CMV-IgM, B19-IgM, biochemistry, CBC, coagulation)
Nadeem et al. (2021) [55]	USA	Case report	Inpatient	1	62 years	EBV coinfection with mild COVID-19 infection	Tool not specified, based on medical records	CT scan, blood tests (biochemistry, serology for EBV viral capsid (VCA) IgM, VCA IgG, EBV EA IgG, EBV NA IgG, coagulation)
Porzionato et al. (2021) [56]	Italy	Case report	Autopsy	1	Not reported	HSV-1 DNA detection and hypopharyngeal ulcers in a deceased COVID-19 patient	RT-PCR	RT-PCR, immunohistochemistry, histologic examination
Shi et al. (2021) [57]	China	Case report	Inpatient	1	66 years	HHV-1 reactivation in a critically ill COVID-19 patient	RT-qPCR	Metagenomic next-generation sequencing, blood test (CBC, biochemistry), Chest X-rays
Wu et al. (2021) [58]	China	Case report	Inpatient	1	45 years	HBV reactivation in COVID-19 patient	RT-PCR	Blood tests (CBC, biochemistry, serology test for HBV surface antigen, HBV surface antibody, HBe Ag, HBe Ab, HBV core antibody), PCR
Xu et al. (2020) [20]	China	Case report	Inpatient (ICU)	1	73 years	Co-reactivations of HSV-1 and VZV in COVID-19 patient	RT-PCR	NGS tests on blood, sputum, and BALF samples, optic bronchoscopy
Yun et al. (2022) [59]	South Korea	Case report	Inpatient	1	18 years	VZV meningitis in COVID-19 patient	RT-PCR	CSF analysis for CBC, CSF PCR
Busani et al. (2021) [60]	Italy	Case series	Inpatient (ICU)	2	Pt 1: 66 years, Pt 2: 49 years	HSV-1 induced acute liver failure in COVID-19 patients	NP swab, tool not specified	Blood tests (biochemistry, coagulation), RT-PCR, CT scan, CSF analysis, histologic examination, chemiluminescence-immunoassay
Hashemi et al. (2020) [61]	Iran	Case series	Inpatient (ICU)	2	Pt 1: 78 years, Pt 2: 75 years	Detection of secondary respiratory pathogens (influenza viruses) in COVID-19 patients with suspected acute respiratory syndrome	NP swab, tool not specified	Blood tests (biochemistry, Influenza virus IgM serology, coagulation)
Kim et al. (2022) [62]	USA	Case series	Retrospective database review	11	Mean age of 59.54 years	Clinical manifestations of patients with SARS-CoV-2 and CMV co-infection	Based on medical records (RT-PCR indicated)	Medical records (CMV PCR indicated)
Moniz et al. (2021) [63]	NA	Case series	Inpatient (ICU)	5	Pt 1: 64 years, Pt 2: 61 years, Pt 3: 61 years, Pt 4: 77 years, Pt 5: 78 years	CMV infection in COVID-19 patients with pneumonia	RT-PCR	CMV PCR (plasma or BAL)
Siddiqui et al. (2022) [64]	India	Case series	Inpatient (ICU)	3	Pt 1: 54 years, Pt 2: 57 years, Pt 3: 62 years	CMV and COVID-19 coinfection	RT-PCR	Blood tests (biochemistry, coagulation, blood smear, CBC), CMV PCR (BAL), bone marrow histologic examination, ophthalmologic examination
Soni et al. (2021) [65]	India	Case series	Ambulatory care	2	Pt 1: 5 years, Pt 2: 61 years	HSV-1 induced ARN in patients with previous history of SARS-CoV-2 infection	RT-PCR	RT-PCR (vitreous sample), ophthalmologic examination
Talan et al. (2022) [66]	Turkey	Case series	Inpatient (ICU)	218	67.7 years (53–84)	CMV reactivation in severe COVID-19 patients	RT-PCR	PCR, CBC
Chen et al. (2021) [67]	China	Cohort	Inpatient	188	36 years (28–52)	Comparison of disease progression between EBV and SARS-CoV-2 co-infection and SARS-CoV-2 alone	RT-PCR	Blood tests (serology for anti-VCA IgM, anti-VCA IgG, anti-EBNA IgG, anti-EA IgM, anti-EA IgM, anti-VCA IgM, biochemistry)
Franceschini et al. (2021) [68]	Italy	Cohort	Inpatient	70	72 years (66–76)	Clinical manifestations from HSV-1 reactivations in COVID-19 patients	RT-PCR	HSV-1 BAL quantitative assay (PCR), blood tests (CBC, biochemistry, cytokine)
Fuest et al. (2022) [69]	Germany	Cohort	Inpatient (ICU)	134	72.5 years (60–78)	Prevalence of HSV-1 and CMV among critically ill COVID-19 patients and the impact of the viruses on patient outcomes, course of disease, and dexamethasone treatment	RT-PCR	CMV and HSV RT-PCR of endotracheal aspirates (ETA), bronchoalveolar lavage (BAL) fluid, and EDTA blood samples, blood tests (CBC, biochemistry)
Gatto et al. (2022) [70]	Italy	Cohort	Inpatient (ICU)	431	65 years (56–72)	Incidence and risk factors of CMV reactivation among critically ill COVID-19 patients	Tool not specified, but indicated as laboratory confirmed	CMV PCR, CT scan, chest X-ray, blood tests (CBC, biochemistry, PCT)
Giacobbe et al. (2021) [71]	Italy	Cohort	Inpatient	41	65 years (60–70)	Cumulative risk of HSV-1 reactivation, its risk factors, and impact on prognosis of critically ill SARS-CoV-2 patients	RT-PCR	HSV-1 BALF quantitative assay (PCR), blood tests (biochemistry, CBC, coagulation)
Hu et al. (2020) [72]	China	Cohort	Inpatient	70	62.8 ± 12.8 years	Comparison of COVID-19 severity and clinical outcomes between patients with and without Influenza A virus infection	RT-PCR	Blood tests (biochemistry, serology)
le balc’h et al. (2020) [15]	France	Editorial letter (cohort)	Inpatient (ICU)	39	64 years (55–72)	HSV and CMV reactivations in COVID-19 patients	RT-PCR	HSV and CMV qRT-PCR, blood tests (CBC)
Lozano et al. (2021) [63]	Spain	Cohort	Inpatient (ICU)	12	37 years (23–50)	Maternal and neonatal outcomes (including CMV reactivations) associated with tocilizumab treatment in pregnant women with severe COVID-19	RT-PCR	Physical examinations, EMR review, blood tests (serology for CMV IgM, IgG, biochemistry, coagulation), CMV PCR
Lino et al. (2022) [34]	Brazil	Cohort	Inpatient	173	52.3 ± 22.9 years	Frequency and clinical impact of HHV-6 coinfection in moderate to critically ill COVID-19 patients	RT-PCR	RT-qPCR, Sanger sequencing, blood test (biochemistry, CBC)
Liu et al. (2020) [35]	China	Cohort	Inpatient	347	Not reported	Comparing liver function changes and disease progression in COVID-19 patients with and without chronic hepatitis B virus infection	Tool not specified, but indicated as admission due to COVID-19	Blood tests (biochemistry, serology for HBsAg), RT-PCR
Meng et al. (2022) [73]	China	Cohort	Inpatient	1314	Not reported	Incidence of EBV reactivation and its impact on the effect of ganciclovir treatment in COVID-19 patients	RT-PCR	Blood tests (biochemistry, CBC, coagulation, ELISA for VCA-IgG, EBV nuclear antigen IgG (EBNA-IgG), VCA-IgM, EA-IgG, CMV-IgG, and CMV-IgM), APACHEII and SOFA scores
Meyer et al. (2021) [74]	France	Cohort	Inpatient (ICU)	153	61.9 years (50.9–70.8)	Impact of HSV reactivation on mortality and HAP/VAP among severe COVID-19 patients	RT-PCR	Blood tests (biochemistry, CBC, coagulation, culture), RT-PCR
Paolucci et al. (2021) [75]	Italy	Cohort	Inpatient (ICU & SICU)	104	ICU: 61.5 years (55–71.25), SICU: 73.5 years (57.8–70)	Opportunistic viral reactivation (EBV reactivation) in COVID-19 patients of varying severity	RT-PCR	Blood test (flow cytometry, chemiluminescent assay)
Peluso et al. (2022) [76]	NA	Cohort (preprint)	Inpatient	280	45 years (35–56)	Comparing EBV prevalence in COVID patients with and without Long COVID symptoms	RT-PCR	qPCR, SIMOA
Saade et al. (2021) [23]	France	Cohort	Inpatient (ICU)	100	60 years (53–67)	Cumulative incidence and risk factors of HSV, EBV, CMV reactivations in severe COVID-19 patients	Not reported	RT-PCR (whole blood, BAL, skin swab) for all viruses, blood tests (CBC, biochemistry)
Seeßle et al. (2021) [77]	Germany	Cohort	Inpatient (ICU)	103	71 years (16)	Frequency and predisposing factors of HSV-1 reactivation in COVID-19 patients	RT-PCR	RT-PCR (BAL or tracheal aspirates) for viral nucleic acid detection and gene expression analysis, FACS for comprehensive immunophenotyping, blood tests (PCT, CBC, biochemistry, cytokine)
Simonnet et al. (2021) [17]	France	Cohort	Inpatient (ICU)	34	58 years (26–81)	Incidence of HHV-6, EBV, and CMV in critically ill COVID-19 patients	RT-PCR	qPCR for all three viruses, chemiluminescence immunoassays
Yang et al. (2022) [78]	China	Cohort	Inpatient (ICU)	2899	71.67 ± 10.07 years	Disease severity and risk factors comparison among SARS-CoV-2 positive patients with different stages of HBV infection	RT-PCR, Chest CT scan	Blood tests (serology test for HBsAg, HBeAg, anti-HBs, anti-HBe, anti-Hbc, biochemistry)
Yue et al. (2020) [33]	China	Cohort	Not reported	307	Influenza A: 61 years (24–68), Influenza B: 56 years (43–66.5)	Clinical characteristics of influenza virus infections in SARS-CoV-2 positive patients	RT-PCR	Blood tests (serology for IAV and IBV IgM, biochemistry)
Zubchenko et al. (2022) [79]	Ukraine	Cohort	Inpatient	88	41.4 ± 6.7 years	Post-COVID manifestations and herpes virus reactivations including EBV, HHV-6, and CMV	Tool not specified, based on medical records	PCR on blood, saliva, and oropharynx samples, ELISA for EBNA-IgG and VCA-IgG, blood tests (CBC, biochemistry, coagulation)
Xie et al. (2021) [22]	China	Cohort	Inpatient	1516	Mean age of 62 years	Clinical outcomes of critically ill COVID-19 patients with EBV reactivation	RT-PCR	EBV RT-PCR, Blood tests (serology for EBV EA IgG and IgM, capsid IgM)
Im et al. (2022) [80]	South Korea	Cross-sectional (relevant to this study)	Inpatient	269	Mean age of 61.6 years	Impact of EBV viremia on COVID-19 severity and prognosis	RT-PCR	EBV RT-PCR, multicolor flow cytometry for lymphocyte subpopulation
Vigón et al. (2021) [81]	Spain	Cross-sectional	Inpatient (ICU)	61	66 years (42–90)	Immunological parameters and herpesvirus reactivations (CMV, EBV) in COVID-19 patients	RT-PCR	ELISA for anti-CMV/IgG, chemiluminescent immunoassay for EBV VCA IgG qPCR for both viruses, ADCC assay

*CMV* cytomegalovirus, *EBV* Epstein Barr virus, *HSV-1, HSV-2* human herpes simplex type 1, 2, *HHV-6, HHV-7* human herpes virus type 6, 7, *IAV* influenza A virus, *IBV* influenza B virus, *VZV* varicella zoster virus, *B19* parvovirus B19, *ADCC* antibody-mediated cytotoxicity assay, *BALF/BAL* bronchoalveolar lavage fluid, *CBC* complete blood count, *CSF* central spinal fluid, *ELISA* enzyme-linked immunosorbent assay, *EMR* electronic medical records, *FACS* fluorescence-activated cell sorting, *NGS* next generation sequencing, *PCR* polymerase chain reaction, *RT-PCR* reverse transcription PCR, *qPCR* quantitative PCR, *ICU* Intensive Care Unit, *IHC* immunohistochemistry, *SICU* Surgical Intensive Care Unit, *PCT* procalcitonin, *APACHEII* Acute Physiology and Chronic Health Evaluation II, *SOFA* Sequential Organ Failure Assessment, *MRI* magnetic resonance imaging, *Ag* antigen, *Ab* antibody, *VCA* viral capsid antigen, IgM immunoglobulin M, *IgG* immunoglobulin G, *EA* early antibody, *NA* nuclear antigen, *HBsAg* hepatitis B surface antigen, *HBeAg* hepatitis B e antigen

^a^Age: Median age with IQR in brackets, mean age with ± SD

^b^Serology: Indicated as general serology test when tool is not specified

### Risk of bias assessment

All cohort studies were categorized as either high quality (n = 13) or moderate quality (n = 8). Stars were deducted from the studies mainly from the Selection domain where the absence of outcome interest, secondary infection, at the start of study was not indicated or the non-COVID-19 cohort was not selected. 11 case reports and 3 series satisfied 70% or more of the CARE checklist items. Majority of the reports were deducted points from the Title domain without a title describing a primary diagnosis or intervention followed by the word “case report”, Abstract and Introduction domains without discussion on scientific benefits of the study, Timeline domain without an organized timeline of patient care, and Patient Perspective domain without discussion on patient’s experience with treatment(s) they received. Some case reports also did not indicate whether the patient consents were obtained. Assessment results are available on Appendix S2 in Additional file 2.

### Definition of reactivation and co-infection

The review identified 40 reactivation studies, 8 studies co-infection studies, and 5 studies where concomitant infection in COVID-19 patients was not distinguished as either reactivation or co-infection. All reported cases have been categorized accordingly to authors’ descriptions in relevant studies and Table 2 summarizes the detection criteria of each virus by type of infection.

**Table 2:** Summary of detection criteria for each infection category

Virus	Reactivation detection criteria	Co-infection detection criteria	Not distinguished
Parvovirus B19	Not applicable	Not applicable	All studies describing an infection interchangeably with the terms “reactivation” and “coinfection” and the detection criteria are the following: Seropositive for B19 IgM [54]
EBV	All studies describing an infection as “reactivation” and the detection criteria are the following: Undetectable viremia from an initial molecular testing followed by the detection of EBV DNA in a subsequent testing [17], or detection of EBV DNA [16, 22, 73, 75, 76, 79, 81] and seropositivity for VCA-IgG and NA-IgG [76, 81], VCA IgG and EA-IgG [73], EA-D IgG and/or with NA IgG [76], VCA-IgG only [81], or VCA-IgG, NA-IgG, EA IgG/IgM [16, 22]	All studies describing an infection as “coinfection” and the detection criteria are the following: Detection of VCA-IgM [67], and/or with VCA-IgG, EA IgG, NA IgG, positive detection of EBV DNA [55]	All studies describing an infection interchangeably with the terms “reactivation” and “coinfection” and the detection criteria are the following: Detection of EBV DNA [80]or seropositive for the VCA-IgM, VCA-IgG, NA IgG and/or with EA IgM [54]
CMV	All studies describing an infection as “reactivation” and the detection criteria are the following: Undetectable viremia from an initial molecular testing followed by the detection of CMV DNA in a subsequent testing [17, 63, 66, 70, 82], or CMV DNA detection and seropositive for either IgG or IgM [41, 51] or both [41], or detection of CMV DNA alone when prior testing for the absence of viremia is not available [15, 23, 42, 44, 60, 62, 81]	All studies describing an infection as “coinfection” and the detection criteria are the following: Detection of CMV DNA, histological evidence of CMV nuclear inclusion bodies in bone marrow [64]	All studies describing an infection interchangeably with the terms “reactivation” and “coinfection” and the detection criteria are the following: Detection of CMV DNA [43, 48] or seropositive for CMV IgG and IgM [54]
HBV	All studies describing an infection as “reactivation” and the detection criteria are the following: Sudden rise in the level of HBV DNA in the subsequent testing [35, 46, 50, 58, 83]or seropositive for HB surface antigen, HB core antigen IgM, HB envelope antibody (not specified) [36]	Not applicable	Not applicable
IAV	Not applicable	All studies describing an infection as “coinfection” and the detection criteria are the following: Seropositive for IAV IgM [33, 72] or positive detection of IAV RNA [61]	Not applicable
IBV	Not applicable	All studies describing an infection as “coinfection” and the detection criteria are the following: Seropositive for IBV IgM [33]	Not applicable
VZV	All studies describing an infection as “reactivation” and the detection criteria are the following: Undetectable viremia from an initial molecular testing followed by the detection of VZV DNA in a subsequent testing [20]	All studies describing an infection as “coinfection” and the detection criteria are the following: Initial detection of VZV prior to confirmed diagnosis of COVID-19 [59]	All studies describing an infection interchangeably with the terms “reactivation” and “coinfection” and the detection criteria are the following: Seropositive for VZV IgM [45]
HHV-1	All studies describing an infection as “reactivation” and the detection criteria are the following: Undetectable viremia from an initial molecular testing followed by the detection of HHV-1 DNA in a subsequent testing [20, 57, 71], detection of HHV-1 DNA [56, 68, 74] and/or seropositive for HHV-1 IgG [60, 69, 77], history of COVID-19 (recovered) prior to HSV-1 detection, or history of COVID-19 [65] and corneal ulcer prior to detection of HSV-1 [19]	Not applicable	Not applicable
HHV-2	All studies describing an infection as “reactivation” and the detection criteria are the following: Detection of HSV-2 DNA and HSV-2 IgG [49]	Not applicable	Not applicable
HHV-6	All studies describing an infection as “reactivation” and the detection criteria are the following: Detection of HHV-6 DNA [16, 17, 79] and/or seropositive for HHV-6 IgG [16]	All studies describing an infection as “coinfection” and the detection criteria are the following: Concomitant detection of HHV-6 DNA with COVID-19 diagnosis [34]	Not applicable
HHV-7	All studies describing an infection as “reactivation” and the detection criteria are the following: Detection of HHV-7 IgG [16]	Not applicable	Not applicable
HHV-8	All studies describing an infection as “reactivation” and the detection criteria are the following: Undetectable viremia from an initial molecular testing followed by the detection of HHV-8 DNA in a subsequent testing [71] or seropositive for HHV-8 antibodies (not specified) following a complete recovery from COVID-19 [52]	Not applicable	Not applicable

*CMV* cytomegalovirus, *EBV* Epstein Barr virus, *HSV-1, HSV-2* human herpes simplex type 1,2, *HHV-6, HHV-7* human herpes virus type 6, 7, *IAV* influenza A virus, *IBV* influenza B virus, *VZV* varicella zoster virus, *B19* parvovirus B19, *Ag* antigen, *Ab* antibody, *VCA* viral capsid antigen, *IgM* immunoglobulin M, *IgG* immunoglobulin G, *EA* early antibody, *NA* nuclear antigen, *HBsAg* hepatitis B surface antigen, *HBeAg* hepatitis B e antigen

#### Reactivation

Table 3 summarizes the findings for the reactivation cohorts. In total, there were 895 cases of reactivations, among which 519 (58%) were Epstein Barr virus (EBV) reactivation, 157 (17.5%) were human herpes virus 1 (HHV-1) reactivation, 154 (17.2%) were cytomegalovirus (CMV) reactivation, 47 (5.3%) were HHV-6 reactivation, 14 (1.6%) were hepatitis B virus (HBV) reactivation, 2 (0.2%) were HHV-8, and 1 (0.11%) of varicella zoster virus (VZV), HHV-2, HHV-7, and HHV-8 were observed reactivations were observed at rates less than 1% each. 7 studies reported 336 patients who underwent co-reactivations of different viruses.

**Table 3:** Summary of clinical findings from reactivations

Virus	EBV	CMV	VZV	HHV-1	HHV-2	HHV-6	HHV-7	HHV-8	HBV	Total (%)
Number of cases	519	154	1	157	1	47	1	2	13	895 (100.00)
Sex										
Female	45	36		29	1			1	5	117 (32.77)
Male	45	93	1	94		1	1	1	4	240 (67.23)
Total	90	129	1	123	1	1	1	2	9	357 (100.00)
COVID-19 severity										
Asymptomatic				1					1	2 (0.52)
Moderate				1						1 (0.26)
Severe/Critical	103	116		150		7			5	381 (99.22)
Total	103	116	0	152	0	7	0	0	6	384 (100.00)
Comorbidities/Medical history										
Asthma		3								3 (1.37)
Arthritis		1								1 (0.46)
Cardiovascular disease		8		12					2	22 (10.05)
Chronic kidney disease		1								1 (0.46)
Chronic renal failure				1						1 (0.46)
Diabetes		37		13				1	2	53 (24.20)
History of transplant		4		2						6 (2.74)
Hypercholesterolemia		1								1 (0.46)
Hypertension		61		3				2	4	70 (31.96)
Hyperlipidemia		5								5 (2.28)
Hyperthyroidism		1								1 (0.46)
Hyperuricemia		1								1 (0.46)
Past HBV infection		1							6	7 (3.20)
HIV		1								1 (0.46)
Immunosuppression (non-specific)		21		17						38 (17.35)
Malignancy		1		1					2	4 (1.83)
Pneumonia		2		1					2	5 (2.28)
Pulmonary fibrosis		2		1						3 (1.37)
Total	0	151	0	51	0	0	0	3	18	219 (100.00)
Clinical manifestations										
Symptomatic										
Cough		5		29						34 (50.00)
Diarrhea		1								1 (1.47)
Dyspnea		1		3						4 (5.88)
Fever	1	4		4		1	1	1		12 (17.65)
Jaundice		4							2	6 (8.82)
Myalgia	1	1				1	1			4 (5.88)
Skin lesions		1	1	1						3 (4.41)
Rash	1					1	1			3 (4.41)
Vomiting									1	1 (1.47)
Total	3	17	1	37	0	3	3	1	3	68 (100.00)
Complications										
Acidosis	1									1 (0.17)
AKI	12									12 (2.04)
ALI									1	1 (0.17)
AMI	2									2 (0.34)
ARDS	108	9							1	118 (20.07)
Cirrhosis									1	1 (0.17)
Gingivostomatitis				2						2 (0.34)
Encephalitis				1						1 (0.17)
Encephalopathy		1								1 (0.17)
Heart failure	1									1 (0.17)
Hemorrhagic esophagitis		1								1 (0.17)
Hepatitis				2						2 (0.34)
Keratitis				1						1 (0.17)
Malignancy								2		2 (0.34)
Renal failure				1						1 (0.17)
Retinal necrosis				2	1					3 (0.51)
Pneumonia		2		1				1		4 (0.68)
Seizure		1								1 (0.17)
Septic shock/sepsis		1	1	1						3 (0.51)
Death	12	16		31					1	60 (10.20)
ICU admission	126	111	1	119		7		1	5	370 (62.93)
Total	262	142	2	161	1	7	0	4	9	587 (100.00)
Laboratory findings										
Leukopenia	1	1				1	1			4 (0.37)
Lymphopenia	1	89		51		1	1		1	144 (13.42)
Neutrophilia		1								1 (0.09)
Thrombocytopenia								1		1 (0.09)
Decreased hemoglobin level				21						21 (1.96)
Decreased prothrombin time				1						1 (0.09)
Elevated ALT level	66	2		2					3	73 (6.80)
Elevated AST level	11	2		2					3	18 (1.68)
Elevated creatinine level				1						1 (0.09)
Elevated CRP level		89		117				1		207 (19.29)
Elevated D-dimer level	55	88		62						205 (19.11)
Elevated ferritin level	55			50						105 (9.79)
Elevated fibrinogen level	55									55 (5.13)
Elevated interleukin-6 level	55			14						69 (6.43)
Elevated LDH level		88		62						150 (13.98)
Elevated procalcitonin level				15						15 (1.40)
Elevated total bilirubin level		1		1					1	3 (0.28)
Total	299	361	0	399	0	2	2	2	8	1073 (100.00)
Pharmacological interventions										
Acyclovir		72	1	111	1					185 (29.13)
Arbidol				1						1 (0.16)
Azithromycin				1				1		2 (0.32)
Betamethasone				2						2 (0.32)
Ceftriaxone		1								1 (0.16)
Cyclosporine		1								1 (0.16)
Dexamethasone		4		39				1		44 (6.93)
Dexmedetomidine				1						1 (0.16)
Everolimus		1								1 (0.16)
Ganciclovir		43			1					44 (6.93)
Heparin		88						1		89 (14.02)
Hydroxychloroquine		2		1						3 (0.47)
Lacosamide		1								1 (0.16)
Levetiracetam		1								1 (0.16)
Meropenem		1								1 (0.16)
Methylprednisolone		2						1	1	4 (0.63)
Midazolam		1		1						2 (0.32)
Morphine				1						1 (0.16)
Mycophenolate mofetil		2								2 (0.32)
Paracetamol				2						2 (0.32)
Prednisolone		9			1					10 (1.58)
Remdesivir		2						1		3 (0.47)
Ribavirin		1							1	2 (0.32)
Ritonavir		2							1	3 (0.47)
Spiramycin		1								1 (0.16)
Tenofovir fumarate									1	1 (0.16)
Tocilizumab		80		8						88 (13.86)
Trimethoprim		1								1 (0.16)
Vancomycin		1								1 (0.16)
Valacyclovir				1						1 (0.16)
Valganciclovir		3								3 (0.47)
Antibiotics (non-specified)								1	2	3 (0.47)
Antivirals (non-specified)									2	2 (0.32)
Steroids (non-specified)		100		23				1	4	128 (20.16)
Total	0	420	1	192	3	0	0	7	12	635 (100.00)
Procedures										
ECMO		3		4						7 (3.33)
Invasive ventilation		89		85						174 (82.86)
Non-invasive ventilation		10		7						17 (8.10)
Renal replacement therapy				6						6 (2.56)
Surgical tracheostomy		2								2 (0.95)
Oxygen support		2		1					1	4 (1.90)
Total	0	106	0	103	0	0	0	0	1	210 (100.00)

*ARDS* acute respiratory distress syndrome, *AKI* acute kidney injury, *ALI* acute liver injury, *AMI* acute myocardial injury, *ALT* alanine transaminase, *AST* aspartate aminotransferase, *CRP* C-reactive protein, *ECMO* extracorporeal membrane oxygenation, *LDH* lactate dehydrogenase, *HBV* hepatitis B virus, *HIV* human immunodeficiency virus, *EBV* Epstein Barr virus, *CMV* cytomegalovirus, *HHV* human Herpes virus, *VZV* varicella zoster virus

Among the cohorts whose COVID-19 severity levels were reported, 103 EBV patients, 116 CMV patients, and 150 HHV-1 patients, and 7 HHV-6 patients populations experienced severe or critical illness. Overall, hypertension (n = 70, 32.0%), immunosuppression (n = 38, 17.4%), diabetes (n = 53, 24.2%), and cardiovascular disease (n = 22, 10.1%) were most frequently observed across reactivation cohorts. In particular, patients with CMV and HHV-1 reactivations had high rates of comorbidities, where hypertension (n = 61, 39.6%), diabetes (n = 37, 2%), immunosuppression (n = 21, 13.6%), and cardiovascular disease (n = 8, 5.2%) were common among CMV patients, and similarly, immunosuppression (n = 17, 12%), diabetes (n = 13, 9.2%), and cardiovascular disease (n = 12, 8.5%) were common among HHV-1 patients. Cough and fever had high frequencies, with fever reported in all populations except in the VZV, HHV-2, and HHV-8 groups. All patient except for the HHV-7 cohort experienced complications. 108 EBV patients and 9 CMV patients reported to have ARDS, taking more than 20% of the complication frequencies.

All patient except for the HHV-7 cohort experienced complications, and death occurred in patients with EBV (n = 12, 2.3%), CMV (n = 16, 6.3%), HHV-1 (n = 31, 19.7%), and HBV (n = 1, 8.3%) reactivations. Among laboratory findings, lymphopenia was observed in all cohorts (n = 144, 13.42%) except for VZV, HHV-2, and HHV-8, and was most prevalent among CMV and HHV-1 patients. Elevated CRP level was the manifestation with the highest overall frequency (n = 207, 19.29%) from CMV (n = 89) and HHV-1 (n = 117) cohorts, next to elevated D-dimer level (n = 205, 19.11%) from EBV (n = 55), CMV (n = 88), HHV-1 (n = 62) cohorts.

When calculating out of total frequencies of pharmaceutical interventions in each virus group, acyclovir (n = 72, 17.1%) and ganciclovir (n = 43, 10.2%) were the most common forms of treatment for CMV reactivation whereas acyclovir alone was provided most frequently for controlling the HSV-1 viremia (n = 111, 57.8%). Overall, steroids (n = 176, 27.7%), including betamethasone (n = 2), dexamethasone (n = 42), methylprednisolone (n = 4), and non-specified steroids (n = 128), as well as tocilizumab (n = 87, 13.9%) were provided as part of the COVID-19 treatment across different virus cohorts.

#### Coinfection

Table 4 summarizes the findings for the coinfection groups. In total, 265 coinfections were reported, among which 187 (70.6%) were IAV, 23 (8.7%) were IBV, 38 (14.3%) were EBV, 13 (4.9%) were HHV-6, 3 (1.1%) were CMV, and 1 (0.04%) was VZV infected patient. None of the cohorts but IAV and CMV reported COVID-19 severity where 10 and 2 patients experienced severe or critical illness, respectively. All but IBV and VZV patient reported comorbidities and past medical history, where cardiovascular disease was observed in both EBV (n = 1) and HHV-6 (n = 8) cohorts whereas diabetes and malignancy were observed in the CMV (n = 2, n = 1, respectively) and HHV-6 (n = 8, n = 4, respectively) cohort.

**Table 4:** Summary of clinical findings from coinfections

Virus	IAV	IBV	EBV	CMV	VZV	HHV-6	Total (%)
Number of cases	187	23	38	3	1	13	265 (100.00)
Sex							
Female	109	12	17			5	143 (53.96)
Male	78	11	21	3	1	8	122 (46.04)
Total	187	23	38	3	1	13	265 (100.00)
COVID severity							
Moderate				1			1 (7.69)
Severe/Critical	10			2			12 (92.31)
Total	10		0	3	0	0	13 (100.00)
Comorbidities/medical history							
Arthritis			1				1 (2.63)
Cardiovascular disease			1			8	9 (23.68)
Chronic lung disease	1						1 (2.63)
COPD				1			1 (2.63)
Diabetes				2		8	10 (26.32)
History of transplant				1			1 (2.63)
Hypertension			1	2			3 (7.89)
Hyperlipidemia			1				1 (2.63)
Immunosuppression (therapeutic)						6	6 (15.79)
Malignancy				1		4	5 (13.16)
Total	1		4	7	0	26	38 (100.00)
Clinical manifestations							
Symptomatic							
Chest pain			1				1 (0.30)
Cough	39	6	28	2			75 (22.87)
Diarrhea	7			1			8 (2.44)
Dyspnea	13	2	1	2			18 (5.49)
Fatigue	24	3					27 (8.23)
Fever	134	16	27	2	1		180 (54.88%)
Myalgia			18		1		19 (5.79%)
Total	217	27	75	7	2	0	328 (100.00%)
Complications							
AKI				1		9	10 (27.78)
AMI				1			1 (27.78)
Encephalopathy				1			1 (27.78)
Melena				1			1 (27.78)
Pneumonia			1	1			2 (5.56)
Septic shock/sepsis				1		9	10 (27.78)
Death	2			2		4	8 (22.22)
ICU admission				3			3 (8.33)
Total	2	0	1	11	0	23	36 (100.00)
Laboratory findings							
Leukopenia						3	3 (1.27)
Lymphopenia	187	23				9	219 (92.80)
Decreased albumin level			1				1 (0.44)
Elevated ALT level			1				1 (0.44)
Elevated AST level			1				1 (0.44)
Elevated CRP level				3			3 (1.27)
Elevated D-dimer level			1	3			4 (1.69)
Elevated ferritin level				2			2 (0.85)
Elevated procalcitonin level			1	1			2 (0.85)
Total	187	23	5	9	0	12	236 (100.00)
Pharmacological interventions							
Acyclovir					1		1 (0.74)
Dexamathasone			1				1 (0.74)
Dexmedetomidine				1			1 (0.74)
Ganciclovir				3			3 (2.21)
Heparin			1				1 (0.74)
Hydroxychloroquine	2						2 (1.47)
Lopinavir/ritonavir	2						2 (1.47)
Oseltamivir	32						32 (23.53)
Piperacillin tazobactam			1				1 (0.74)
Remdesivir			1	1			2 (1.47)
Vancomycin			1				1 (0.74)
Antibiotics (non-specified)			32	1			33 (24.26)
Antivirals			32				32 (23.53)
Steroids			22	2			24 (17.65)
Total	36	0	91	8	1	0	136 (100.00)
Procedures							
Invasive ventilation	1			2			3 (23.08)
Renal replacement therapy				1			1 (7.69)
Supportive oxygen care	2		7				9 (69.23)
Total	3	0	7	3	0	0	13 (100.00)

*AKI* acute kidney injury, *AMI* acute myocardial injury, *ALT* alanine transaminase, *AST* aspartate aminotransferase, *CRP* C-reactive protein, *COPD* chronic obstructive pulmonary disease, *HIV* human immunodeficiency virus, *EBV* Epstein Barr virus, *CMV* cytomegalovirus, *IAV* influenza A virus, *IBV* influenza B virus, *HHV* human herpes virus, *VZV* varicella zoster virus

Out of total, cardiovascular disease and diabetes reported the highest frequencies with 23.68% and 26.32%, respectively. Acute kidney injury and sepsis occurred as complications among the CMV (n = 1, n = 2, respectively) and HHV-6 patients (n = 9, n = 4, respectively). Fever was reported in IAV (n = 134), IBV (n = 16), EBV (n = 27), CMV (n = 2), and VZV (n = 1) cohorts, cough in IAV (n = 39), IBV (n = 6), EBV (n = 28) and CMV (n = 2) cohorts, dyspnea in IAV (n = 13), IBV (n = 2), EBV (n = 1) and CMV (n = 2) cohorts, and myalgia in EBV (n = 18) and VZV (n = 1) cohorts. Among laboratory findings, lymphopenia was dominant in IAV cohort (n = 187), marking the highest frequency with 92.8%. Elevated D-dimer and elevated procalcitonin levels were reported in both EBV (n = 1, n = 1) and CMV (n = 3, n = 1) populations summing to the frequencies of 1.69% and 0.85% each.

For pharmacological interventions, EBV and CMV cohorts had overlaps in receiving remdesivir (n = 1, n = 1, respectively), non-specified antibiotics (n = 32, n = 1, respectively), and steroids (n = 22, n = 2, respectively). Overall, antivirals, including acyclovir, ganciclovir, lopinavir/ritonavir, oseltamivir, remdesivir, and non-specified antivirals, were the most frequently prescribed medications across the cohorts summing up to 53.68% of total interventions, among which oseltamivir was reported with the highest frequency (23.53%). Oxygen therapy and invasive ventilation were common procedures with 69.23% and 23.08% of frequencies.

#### Indistinguishable infection

Table 5 summarizes the findings for indistinguishable groups where the terms “reactivation” and “coinfection” were interchangeably used in articles to describe the patients. In total, 51 cases were indistinguishable. EBV cases (n = 46) were most frequently reported next to CMV (n = 3), VZV (n = 1), and B19 (n = 1). Information on COVID-19 severity was not available on the observed cohorts but EBV, where 7 patients experienced severe or critical illness.

**Table 5:** Summary of clinical findings from indistinguishable infections

	EBV	CMV	VZV	B19	Total (%)
Number of cases	46	3	1	1	51 (100.00)
Sex					
Female	1	3		1	5 (83.33)
Male			1		1 (16.67)
Total					6 (100.00)
COVID					
Severe/Critical	7				7 (100.00)
Total	7	0	0	0	7 (100.00)
Comorbidities/medical history					
Chickenpox			1	1	2 (50.00)
Pneumonia		1		1	2 (50.00)
Total	0	1	1	2	4 (100.00)
Clinical manifestations					
Symptomatic					
Diarrhea			1		1 (25.00)
Rash	1			1	2 (50.00)
Skin lesions			1		1 (25.00)
Total	1	0	2	1	4 (100.00)
Complications					
Neuralgia			1		1 (50.00)
Rectal ulcer		1			1 (50.00)
Total	0	1	1	0	2 (100.00)
Laboratory findings					
Elevated ALT level	1			1	2 (28.57)
Elevated AST level				1	1 (14.29)
Elevated D-dimer level	1			1	2 (28.57)
Elevated LDH level	1			1	2 (28.57)
Total	3	0	0	4	7 (100.00)
Pharmacological interventions					
Acyclovir			1		1 (33.33)
Pregabalin			1		1 (33.33)
Valganciclovir		1			1 (33.33)
Total	0	1	2	0	3 (100.00)
Procedures					
ECMO			1		1 (11.11)
Oxygen support	7	1			8 (88.99)
Total	7	1	1	0	9

*B19* human parvovirus B19, *ALT* alanine transaminase, *AST* aspartate aminotransferase, *CRP* C-reactive protein, *EBV* Epstein Barr virus, *CMV* cytomegalovirus, *ECMO* extracorporeal membrane oxygenation, *VZV* varicella zoster virus

There was no comorbidity or medical history among patient groups except for the history of previous VZV infection in VZV and B19 groups and pneumonia in CMV and B19 groups. 1 EBV and 1 B19 patients reported rash, and 1 CMV and 1 VZV patients underwent rectal ulcer and neuralgia respectively as complications upon infections.

Both EBV and B19 cohorts experienced elevated ALT (n = 1, n = 1), D-dimer (n = 1, n = 1), and LDH levels (n = 1, n = 1). Available treatment information indicates provision of acyclovir and pregabalin to 1 VZV patient each, and valganciclovir for 1 CMV patient for controlling the viral load. Supportive oxygen therapy was most frequent, taking up 88.99% of total procedures.

## Discussion

Overall, our review identified 53 full text articles from the literature search, of which 40 studies reported viral reactivations, 8 studies reported viral coinfections during COVID-19 infection, and 5 studies reported viral infections which were not distinguished as either reactivation or coinfection. Case reports and cohort studies consisted over 50% of the included studies. Among the patients reported, those admitted to the inpatient units including ICU were most common next to the patients receiving the ambulatory care. Risk of bias assessments revealed 13 high and 8 moderate quality cohort studies, 2 high quality cross-sectional studies, and 11 case reports and 3 case series satisfying 70% or more of the CARE checklist items. We did not include any study with prevalence rates available only as one of the primary objectives of this study was to present summary of clinical characteristics of patients. In total, we were able to extract data for 12 viruses including the IAV, IBV, EBV, CMV, VZV, HHV-1, HHV-2, HHV-6, HHV-7, HHV-8, HBV, and Parvovirus B19. Upon summarizing the clinical findings for each infection category, EBV, HHV-1, and CMV were most frequently observed within the reactivation cohort, IAV and EBV within the coinfection cohort, and EBV within the indistinguishable infection cohort. In both reactivation and coinfection groups, patients were commonly reported to have cardiovascular disease, diabetes, and immunosuppression as comorbidities, cough, fever, and myalgia as symptoms, acute kidney injury as complication, and lymphopenia and elevated D-dimer and CRP levels from blood tests. Common pharmaceutical interventions in both groups were steroids including dexamethasone and non-specified steroids, as well as antivirals including acyclovir, ganciclovir, remdesivir, and otherwise non-specified antivirals.

We provide two possible hypotheses for the latent virus reactivation and coinfection mechanisms. First, viral reactivation and coinfection may be attributed to impaired cellular immunity by the existing SARS-CoV-2 infection [75, 78, 84–87]. From our review, we observed high frequencies of lymphopenia with 13.42% and 92.8% in reactivation and coinfection cohorts, respectively. Similarly, retrospective study in China laboratory-confirmed 24 additional respiratory pathogens among COVID-19 patients, and 94.2% of the patients were co-infected with one or more pathogens [84]. Additionally, Paolucci et al. observed a significant loss of lymphocytes in COVID-19 patients and reported an association with the reduction of natural killer (NK) cell and CD8+ T cells and presence of EBV DNA [75]. Exhaustion of functional T cells have been observed among COVID-19 patients marked by the increased expressions of the programmed cell death protein 1 (PD-1) and T cell immunoglobulin and mucin domain 3 (Tim-3), which are indicative of the loss of effector T cell function in viral clearance [78, 85, 86]. Additionally, Ouyang et al. in their 2020 reported the downregulation of the key proteins in TCR signaling and T-cell activation, including SOS1 and MAP2K7, during SARS-CoV-2 infection [87]. Although there are accumulating evidence of diminished immunity among COVID-19 patients, specific immunopathological mechanisms of COVID-19 and viral reactivations and coinfections are yet to be established.

Second, majority of patients who were critically ill from the SARS-CoV-2 infection upon admission received immunomodulatory agents. Steroidal inhibition of inflammatory proteins through gene suppression and blockade of T cell differentiation and monoclonal antibody blockade of IL-6 receptor have been proposed to be beneficial for controlling the cytokine storm and associated organ damage [88–90], yet such immunosuppressant properties of treatment may had favored opportunistic infections or latent virus replications [91, 92]. This is supported by the evidence of infections among the patients treated with tocilizumab (TCZ), for example, where studies reported the occurrence of infections as the most common side effect in patients with rheumatoid arthritis treated with TCZ [93], as well as the occurrence of herpes infections among COVID-19 patients who received high doses of glucocorticoids after TCZ administration [94]. In our review, steroids, including non-specified steroids, dexamethasone, prednisolone, and methylprednisolone, and tocilizumab contributed 18.11% and 13.86% of the CMV cohort medications, respectively—Gatto et al. additionally reported bacterial infections among COVID-19 patients who received steroids during ICU stay, also suggesting the immunomodulatory role of medications in activating virus latency [70]. However, there are conflicting evidence to whether the pharmacologic immunosuppression directly contributes to increasing the rates of infectious disease complications as impaired host immunity from a natural course of SARS-CoV-2 infection, comorbidities prior to treatment, and most importantly, administration of varying doses at different time points should also be taken into account.

Compared to a large body of literature focusing on a specific virus, we undertook a comprehensive review of all viral infections observed in COVID-19 patients, which enabled us to collect comparative clinical findings between different viruses. Our findings indicate that EBV, CMV, and HHV-6 were most either reactivated or co-occurred upon SARS-CoV-2 infection. We’ve also distinguished observed cases by reactivation and coinfection categories based on authors’ descriptions of detection criteria, which are described in Table 2, which may provide additional clarity on the status of patient’s infectious complication. Overall, evidence collected in this review indicate a potential risk of latent virus reactivation or secondary infection among COVID-19 patients and the cautious use of immunomodulatory agents. Further, high degree of similarity in symptoms between the single SARS-CoV-2 infection and coinfections with other pathogens, especially those of IAV and IBV, might imply that timely detection of reactivated and coinfected viruses is necessary to prevent severe illness and complications. However, based on the previous evidence of viral reactivations among the patients of other critical conditions such as cancer, longer ICU stay, severe pneumonia, or sepsis, imply more of a casual than absolute association between COVID-19 and detection of latent viral DNA. Thus, it is necessary to elaborate that the COVID-19 is rather an additional mechanism to other clinical conditions that may trigger viral reactivations and co-infections [95–98].

There were several limitations in this study associated with the availability of information from the selected articles. First, there were challenges in differentiating between the reactivation and coinfection of viruses as some of authors interchangeably used the terms to describe an infection or did not provide accurate detection criteria used in study besides describing an infection as either reactivation or coinfection within the text or title of article. Second, demographic information of patients of our interest was not available in all studies, which limited the extent of comparisons between different virus groups. Some cohort studies, for example, did not provide the sex ratio of population or provided the sex ratio of the entire cohort including the patients with and without reactivations or coinfections but not the target of our interest. Out of 50 extracted articles, only 9 studies provided ethnicity of patients (not shown). Third, considering the longevity of viral IgG presence upon infection that could range from several years to lifelong time upon infection [99–102], diagnostic approach with IgG detection alone in some of the studies in review may had overestimated the occurrences of viral reactivation. Lastly, reference ranges for laboratory findings either varied by studies or were not available, although the thresholds for high, normal, and low levels of each biomarker overlapped.

## Conclusion

The study showed that patients were reported to have risk factors of severe COVID-19 including cardiovascular disease, diabetes, and immunosuppression as comorbidities, as well as additionally disease aggravating conditions including acute kidney injury as complication, lymphopenia and elevated D-dimer and CRP levels from laboratory testing, and steroids and antivirals for treatment [103–107]. Further, EBV, HHV-1, and CMV were most frequently observed within the reactivation cohort, IAV and EBV within the coinfection cohort, and EBV within the indistinguishable infection cohort. However, it is important to note that our study did not attempt to imply an increased risk of infection among individuals with latent viruses. Through this systematic review, we aimed to collect and summarize the available evidence to fulfill the literature gap on the clinical outcomes and potential risk factors of both reactivations and coinfections. Further, our review highlights a significant need for standardizing the detection criteria for reactivation and coinfection, especially within the context of comparing cases across different study sites. With the complex immune pathways triggered by SARS-CoV-2 exposing the patients to develop more adverse outcomes from other concurrent or latent infections, it is imperative that more scientific evidence become available for more efficient treatment and diagnostic measures.

## Supplementary Information

**Additional file 1.** PRISMA 2020 Checklist.

**Additional file 2: Appendix S1.** Search terms for each database. Appendix S2. Risk of bias assessments.

## Data Availability

All data generated or analysed during this study are included in this published article (Additional file 2).

## Abbreviations

AKI: Acute kidney injury
ALI: Acute liver injury
AMI: Acute myocardial injury
APACHEII: Acute Physiology and Chronic Health Evaluation II
ALT: Alanine transaminase
Ag: Antigen
Ab: Antibody
ADCC: Antibody-mediated cytotoxicity assay
AST: Aspartate aminotransferase
BALF/BAL: Bronchoalveolar lavage fluid
B19: Human parvovirus B19
CBC: Complete blood count
CRP: C-reactive protein
CSF: Central spinal fluid
COPD: Chronic obstructive pulmonary disease
CT: Computed tomography
CARE: Consensus-based Clinical Case Reporting
COVID-19: Coronavirus disease 2019
CMV: Cytomegalovirus
EA: Early antibody
ELISA: Enzyme-linked immunosorbent assay
EMR: Electronic Medical Records
EBV: Epstein Barr virus
ECMO: Extracorporeal membrane oxygenation
FACS: Fluorescence-activated cell sorting
HBV: Hepatitis B virus
HBsAg: Hepatitis B surface antigen
HBeAg: Hepatitis B e antigen
HSV-1: Human herpes simplex type 1
HSV-2: Human herpes simplex type 2
HSV-6: Human herpes simplex type 6
HSV-7: Human herpes simplex type 7
HIV: Human immunodeficiency virus
IAV: Influenza A virus
IBV: Influenza B virus
ICU: Intensive Care Unit
IHC: Immunohistochemistry
IL-2: Interleukin-2
IgG: Immunoglobulin G
IgM: Immunoglobulin M
LDH: Lactate dehydrogenase
MAP2K7: Mitogen-activated protein kinase kinase
MRI: Magnetic resonance imaging
NA: Nuclear antigen
NGS: Next Generation Sequencing
NOS: Newcastle–Ottawa Scale
PCR: Polymerase chain reaction
PCT: Procalcitonin
PRISMA: Preferred Reporting Items for Systematic Reviews and Meta-Analyses
PD-1: Programmed death 1
qPCR: Quantitative PCR
RT-PCR: Reverse transcription PCR
SOFA: Sequential Organ Failure Assessment
SOS1: Son of sevenless homolog 1
SICU: Surgical Intensive Care Unit
TCR: T cell receptor
TCZ: Tocilizumab
TIM-3: T cell immunoglobulin and mucin domain 3
TNF: Tumor Necrosis Factor
VCA: Viral capsid antigen
VZV: Varicella zoster virus
