# Supplementary material for: Viral reactivations and co-infections in COVID-19 patients: a systematic review
Source: BMC Infect Dis. 2023 Apr 26;23:259. doi: 10.1186/s12879-023-08117-y (PMC10131452; doi:10.1186/s12879-023-08117-y)
Supplement: Supplementary file 2 — Additional file 2: Appendix S1. Search terms for each database. Appendix S2. Risk of bias assessments. [file 12879_2023_8117_MOESM2_ESM.docx]

**Appendix S1: Search terms for each database**

**PUBMED**

(Viruses [mh] OR viruses [tw] OR Virus [All Fields] OR viral [All Fields] OR Retroviridae [mh] OR Retrovirus [all fields] OR Herpesviridae OR “Zoster”[All Fields] OR “Herpes zoster”[MeSH Terms] OR “Herpes zoster”[All Fields] OR “Shingles”[All Fields] OR “VZV”[All Fields] OR Herpesviridae [mh] OR Herpes Simplex [mh] OR herpes [all fields] OR Epstein-Barr Virus Infections [mh] OR Epstein-Barr virus [all fields] OR herpesvirus [all fields] OR cytomegalovirus [mh] OR cytomegalovirus [all fields] OR Hepatitis, Viral, Human [mh] OR HBV [All Fields] OR BK Polyomavirus OR BK virus [mh]) **AND** (“COVID-19”[MeSH Terms] OR “COVID-19”[All Fields] OR “Coronavirus disease 2019”[All Fields] OR “SARS-CoV-2”[MeSH Terms] OR “SARS-CoV-2”[All Fields]) AND (Latent Infection [mh] OR latent [tw] OR reactivation [all fields] OR Reactivated [all fields] OR resurrection [all fields])

**EMBASE**

(‘Zoster’/exp OR Zoster OR ‘Herpes zoster’/exp OR Herpes zoster OR ‘Shingles’/exp OR Shingles OR ‘VZV’/exp OR VZV OR ‘Herpesviridae’/exp OR Herpesviridae OR ‘Herpes Simplex’/exp OR Herpes Simplex OR ‘herpes’/exp OR herpes OR ‘herpesvirus’/exp OR herpesvirus OR ‘Epstein-Barr Virus’/exp OR Epstein-Barr Virus OR ‘Epstein-Barr Virus Infections’/exp OR Epstein-Barr Virus Infections OR ‘cytomegalovirus’/exp OR cytomegalovirus OR ‘Hepatitis’/exp OR Hepatitis OR ‘HBV’/exp or HBV OR ‘BK Polyomavirus’/exp OR BK Polyomavirus OR ‘BK virus’/exp OR BK virus OR ‘Retroviridae’/exp OR Retrovirus) AND (‘COVID-19’/exp OR COVID-19 OR ‘Coronavirus disease 2019’/exp OR Coronavirus disease 2019 OR ‘SARS-CoV-2’/exp OR SARS-CoV-2) AND (‘Latent Infection’/exp OR Latent Infection OR ‘latent’/exp OR latent OR ‘reactivation’/exp OR reactivation OR ‘Reactivated’/exp OR Reactivated OR ‘resurrection’/exp OR resurrection)

**Google Scholar**

Latent virus reactivation in COVID-19 patients, Latent virus resurrection in COVID-19 patients, Virus reactivation in COVID-19 patients, Virus resurrection in COVID-19 patients, Latent virus reactivation in SARS-CoV-2, Latent virus reactivation and SARS-CoV-2, Virus reactivation in SARS-CoV-2, Virus reactivation and SARS-CoV-2, Virus reactivation in SARS-CoV-2, Virus resurrection and SARS-CoV-2

**medRxiv**

(Zoster OR Herpes Zoster OR Shingles OR VZV OR OR Retrovirus OR Retroviridae OR Herpes Simplex OR herpes OR Epstein-Barr Virus OR Epstein-Barr Virus Infection OR EBV OR cytomegalovirus OR Hepatitis OR Hepatitis B OR Polyomavirus OR BK Polyomavirus) AND (Latent virus OR Latency) AND (reactivation OR resurrection OR Reactivation OR Resurrection) AND (SARS-CoV-2 OR COVID-19 OR Coronavirus disease 2019)

**Appendix S2: Risk of bias assessments**

***Table S1. Quality assessment for cohort studies using NOS scale***

| **Author, year** | **Selection** | | | | **Comparability** | **Outcome** | | | **Total** |
| --- | --- | --- | --- | --- | --- | --- | --- | --- | --- |
|  | **1) Representativeness of the exposed cohort** | **2)**  **Selection of the non exposed cohort** | **3) Ascertainment of exposure** | **4) Outcome of interest was not present at start** | **1) Comparability of cohorts on the basis of the design or analysis** | **1) Assessment of outcome** | **2)**  **Long follow up for outcomes to occur** | **3) Adequacy of follow up** |  |
| Chen et al, 2021 | 1 | 1 | 1 | 0 | 2 | 0 | 0 | 0 | 5 |
| Franceschini et al, 2021 | 1 | 0 | 1 | 0 | 2 | 1 | 1 | 1 | 7 |
| Fuest et al, 2022 | 1 | 1 | 1 | 0 | 0 | 1 | 1 | 1 | 6 |
| Gatto et al, 2022 | 1 | 1 | 1 | 1 | 2 | 1 | 1 | 1 | 9 |
| Giaccobe et al, 2021 | 1 | 0 | 0 | 1 | 1 | 1 | 1 | 1 | 5 |
| Hu et al, 2020 | 1 | 1 | 1 | 0 | 1 | 1 | 1 | 1 | 7 |
| Le balch’h et al, 2020 | 1 | 0 | 1 | 1 | 1 | 1 | 1 | 1 | 7 |
| Lino et al, 2022 | 1 | 1 | 1 | 0 | 2 | 1 | 1 | 1 | 8 |
| Liu et al, 2020 | 1 | 1 | 1 | 1 | 2 | 1 | 1 | 1 | 9 |
| Lozano et al, 2020 | 1 | 0 | 1 | 0 | 0 | 1 | 1 | 1 | 5 |
| Meng et al, 2022 | 1 | 1 | 1 | 0 | 1 | 1 | 1 | 1 | 7 |
| Meyer et al, 2022 | 1 | 1 | 1 | 1 | 2 | 1 | 1 | 1 | 9 |
| Paolucci et al, 2021 | 1 | 1 | 1 | 0 | 1 | 1 | 0 | 1 | 6 |
| Peluso et al, 2022 | 1 | 1 | 1 | 0 | 1 | 1 | 0 | 0 | 5 |
| Saade et al, 2021 | 1 | 0 | 1 | 1 | 1 | 1 | 1 | 1 | 7 |
| Seeble et al, 2021 | 1 | 1 | 1 | 1 | 1 | 0 | 1 | 1 | 7 |
| Simmonet et al, 2021 | 1 | 0 | 1 | 0 | 0 | 1 | 1 | 1 | 5 |
| Xie et al, 2021 | 1 | 1 | 1 | 0 | 1 | 1 | 1 | 1 | 7 |
| Yang et al, 2022 | 1 | 0 | 0 | 0 | 1 | 1 | 1 | 1 | 5 |
| Yue et al, 2020 | 1 | 1 | 1 | 0 | 1 | 1 | 1 | 1 | 7 |
| Zubchenko et al, 2022 | 1 | 1 | 1 | 1 | 1 | 1 | 1 | 1 | 8 |

***Table S2. Quality assessments for case series and case studies using CARE checklist***

| **Author** | **1)**  **Title** | **2)**  **Key Words** | **3)**  **Abstract** | **4)**  **Introduction** | **5)**  **Patient Information** | **6)**  **Findings** | **7)**  **Timeline** | **8)**  **Diagnostic assessment** | **9)**  **Intervention** | **10) Follow-up and outcomes** | **11, 12)**  **Discussion and Patient perspective** | **13)**  **Informed consent** | **Total** |
| --- | --- | --- | --- | --- | --- | --- | --- | --- | --- | --- | --- | --- | --- |
| Aldhaleei et al, 2020 | 1 | 1 | 4 | 0 | 3 | 1 | 0 | 3 | 2 | 2 | 3 | 0 | 20 |
| Aldehaim et al, 2022 | 1 | 1 | 4 | 0 | 4 | 1 | 0 | 3 | 3 | 3 | 3 | 1 | 24 |
| Ananthegowda et al, 2020 | 1 | 1 | 2 | 0 | 3 | 1 | 0 | 3 | 3 | 3 | 3 | 1 | 21 |
| Busani et al, 2021 | 0 | 1 | 2 | 0 | 3 | 1 | 1 | 3 | 3 | 3 | 3 | 1 | 21 |
| Carll et al, 2021 | 0 | 1 | 4 | 1 | 3 | 1 | 0 | 3 | 3 | 3 | 4 | 1 | 24 |
| Das et al, 2022 | 1 | 1 | 4 | 0 | 4 | 1 | 0 | 3 | 2 | 2 | 3 | 1 | 22 |
| Drago et al, 2020 | 0 | 1 | 3 | 0 | 2 | 1 | 0 | 3 | 0 | 2 | 3 | 0 | 15 |
| Duong et al, 2021 | 0 | 1 | 0 | 1 | 2 | 1 | 0 | 3 | 1 | 2 | 3 | 0 | 14 |
| Ferreira et al, 2020 | 0 | 1 | 3 | 0 | 4 | 1 | 0 | 3 | 2 | 2 | 3 | 1 | 20 |
| Figueredo et al, 2021 | 1 | 1 | 0 | 1 | 3 | 1 | 0 | 3 | 2 | 2 | 3 | 0 | 17 |
| Gardini et al, 2021 | 1 | 1 | 4 | 1 | 4 | 1 | 0 | 4 | 3 | 3 | 3 | 1 | 26 |
| Ghobrial et al, 2021 | 0 | 1 | 0 | 1 | 4 | 1 | 0 | 3 | 2 | 2 | 3 | 0 | 17 |
| Gonzalez et al, 2021 | 0 | 1 | 3 | 0 | 3 | 1 | 0 | 3 | 3 | 2 | 3 | 1 | 20 |
| Hashemi et al, 2020 | 0 | 1 | 0 | 0 | 2 | 1 | 0 | 3 | 1 | 3 | 2 | 0 | 13 |
| Jimenez et al, 2021 | 1 | 1 | 1 | 0 | 4 | 1 | 0 | 3 | 2 | 3 | 2 | 0 | 18 |
| Khatib et al, 2020 | 0 | 1 | 3 | 1 | 3 | 1 | 0 | 3 | 3 | 3 | 3 | 1 | 22 |
| Kim et al, 2022 | 1 | 1 | 2 | 0 | 4 | 1 | 0 | 2 | 2 | 1 | 3 | 1 | 18 |
| Magri et al, 2021 | 0 | 1 | 3 | 0 | 4 | 1 | 0 | 3 | 3 | 2 | 3 | 1 | 21 |
| Maillet et al, 2021 | 0 | 1 | 3 | 0 | 2 | 1 | 0 | 3 | 3 | 3 | 3 | 1 | 20 |
| Mikami et al, 2021 | 0 | 1 | 0 | 1 | 3 | 1 | 0 | 3 | 0 | 2 | 4 | 0 | 15 |
| Moniz et al, 2021 | 1 | 1 | 0 | 0 | 4 | 1 | 0 | 2 | 2 | 2 | 4 | 1 | 18 |
| Nadeem et al, 2021 | 0 | 1 | 1 | 0 | 4 | 1 | 0 | 3 | 3 | 3 | 4 | 1 | 21 |
| Porzionato et al, 2021 | 1 | 1 | 4 | 1 | 3 | 1 | 1 | 3 | 2 | 3 | 4 | 1 | 25 |
| Shi et al, 2021 | 1 | 1 | 3 | 1 | 3 | 1 | 0 | 3 | 2 | 2 | 3 | 1 | 21 |
| Siddiqui et al, 2022 | 0 | 1 | 3 | 1 | 4 | 1 | 0 | 3 | 3 | 3 | 3 | 0 | 22 |
| Soni et al, 2021 | 0 | 1 | 3 | 1 | 4 | 1 | 0 | 3 | 2 | 3 | 3 | 0 | 21 |
| Talan et al, 2022 | 0 | 0 | 0 | 1 | 2 | 1 | 0 | 2 | 1 | 2 | 3 | 1 | 13 |
| Wu et al, 2021 | 1 | 1 | 2 | 0 | 4 | 1 | 0 | 3 | 2 | 1 | 2 | 1 | 18 |
| Xu et al, 2020 | 0 | 1 | 0 | 1 | 2 | 1 | 1 | 3 | 2 | 2 | 3 | 0 | 15 |
| Yun et al, 2022 | 1 | 1 | 2 | 1 | 3 | 1 | 1 | 3 | 2 | 3 | 3 | 1 | 22 |

***Table S3. Quality assessment for cross-sectional studies using adapted NOS scale***

| **Author** | **Selection** | | | | **Comparability** | **Outcome** | | **Total** |
| --- | --- | --- | --- | --- | --- | --- | --- | --- |
|  | **1) Representativeness of the sample** | **2)**  **Sample size** | **3)**  **Non-respondents** | **4) Ascertainment of the exposure** | **1) The subjects in different outcome groups are comparable, based on the study design**  **or analysis. Confounding factors are controlled.** | **1) Assessment of the outcome** | **2)**  **Statistical test** |  |
| Im et al, 2022 | 1 | 1 | 1 | 2 | 2 | 2 | 1 | 10 |
| Vignon et al, 2021 | 1 | 1 | 1 | 2 | 0 | 2 | 1 | 8 |
